# Neutrophil extracellular traps in asthma and chronic obstructive pulmonary disease: pathogenic roles, and therapeutic opportunities

**DOI:** 10.3389/fimmu.2026.1919974

**Published:** 2026-09-07

**Authors:** Xijie Zhang, Tobias Dallenga, Susanne Krauss-Etschmann

**Affiliations:** 1 Division of Early Life Origins of Chronic Lung Diseases, Research Center Borstel (RCB), Airway Research Center North (ARCN), German Center for Lung Research (DZL), Borstel, Germany; 2 Division of Cellular Microbiology, Research Center Borstel-Leibniz Lung Center, Borstel, Germany; 3 German Center for Infection Research (DZIF), Partner Site Hamburg-Lübeck-Borstel-Riems, Borstel, Germany; 4 DZL-Laboratory for Experimental Microbiome Research, Research Center Borstel, Airway Research Center North (ARCN), German Center for Lung Research (DZL), Borstel, Germany; 5 Institute of Experimental Medicine, Christian-Albrechts University Kiel, Kiel, Germany

**Keywords:** asthma, chronic obstructive pulmonary disease (COPD), neutrophil extracellular traps (NETs), T cells, therapy

## Abstract

Asthma and chronic obstructive pulmonary disease (COPD) are the most prevalent chronic inflammatory airway diseases and constitute a substantial global health burden. Neutrophils are key effector cells of the innate immune system, playing an important role in the pathogenesis of multiple respiratory diseases. Recently, increasing attention has focused on the formation of neutrophil extracellular traps (NETs) as an important mechanism in diverse disease contexts, including neutrophilic asthma and COPD. NETs are web-like structures composed of DNA and diverse granule proteins that can trap and neutralize pathogens and modulate inflammatory responses. However, NETs also contain several cytotoxic proteins that can cause tissue damage, impair microbial clearance and promote disease progression. In this review, we summarize the components of NETs, the molecular mechanisms underlying NET formation, the interplay between NETs and T cells, current findings on the role of NETs in asthma and COPD, and emerging NET-targeted therapeutic strategies. Finally, we highlight current knowledge gaps and discuss future research directions in the field of NETs in asthma and COPD.

## 1 Introduction

Asthma and chronic obstructive pulmonary disease (COPD) are major chronic inflammatory airway diseases (1, 2) characterized by persistent airway inflammation, airway remodeling and airflow limitation. Although both diseases share features of chronic airway inflammation, they differ in their underlying pathophysiology and clinical manifestations. Asthma is characterized by airway hyperresponsiveness (AHR) with reversible airflow obstruction, whereas airflow limitation in COPD is largely irreversible and progressive.

Clinically, asthma presents with recurrent symptoms including wheezing, coughing, shortness of breath and chest tightness. It affects approximately 260 million people worldwide (1, 3) and comprises several heterogeneous endotypes that are commonly classified by the dominant granulocyte populations in bronchioalveolar lavage fluid (BALF) or induced sputum (4). These include predominantly eosinophilic, neutrophilic (5), mixed-granulocyte or paucigranulocytic endotypes (6). Among these, neutrophilic asthma is frequently associated with severe disease and poorer responsiveness to corticosteroids (GCs) and other controller medications (7).

According to the WHO, COPD was the fourth leading cause of death worldwide in 2021 (8). The disease is characterized by emphysematous alveolar destruction, chronic obstructive bronchitis, increased neutrophil infiltration and recurrent acute exacerbations triggered by bacterial or viral infections (2, 9). Elevated blood neutrophil counts are associated with an increased risk of exacerbation and mortality in patients with COPD (10).

Together, these observations highlight the important role of neutrophilic inflammation in both asthma (11) and COPD (10).

In response to infection or tissue injury, including exposure to cigarette smoke, airway epithelial cells, macrophages, and other resident cells release neutrophil-attracting chemokines such as CXCL1, CXCL2, CXCL5, and CXCL8, initiating the mobilization of neutrophils from the bone marrow and their subsequent recruitment into the lung (12–15). Once recruited to the airways, neutrophils contribute to innate host defense through phagocytosis, degranulation, cytokine production, and the formation of neutrophil extracellular traps (NETs) (16–18). NETs are web-like structures that facilitate pathogen trapping and neutralization. Their primary components are chromatin complexes of cell-free DNA (cfDNA), citrullinated histones and several neutrophilic granule-derived proteins, including myeloperoxidase (MPO), neutrophilic elastase (NE) (19), cathepsin G (CathG) and members of the matrix metalloproteinase (MMP) family. NETs participate in both innate and adaptive immune responses (20–22). Depending on the biological context, NETs can either contribute to immune homeostasis or promote pathological immune dysregulation. Excessive or dysregulated NET formation has been implicated in numerous human diseases such as sterile inflammatory disorders, autoimmune diseases, thrombosis, pregnancy related complications and cancer (22).

**Figure 1:**
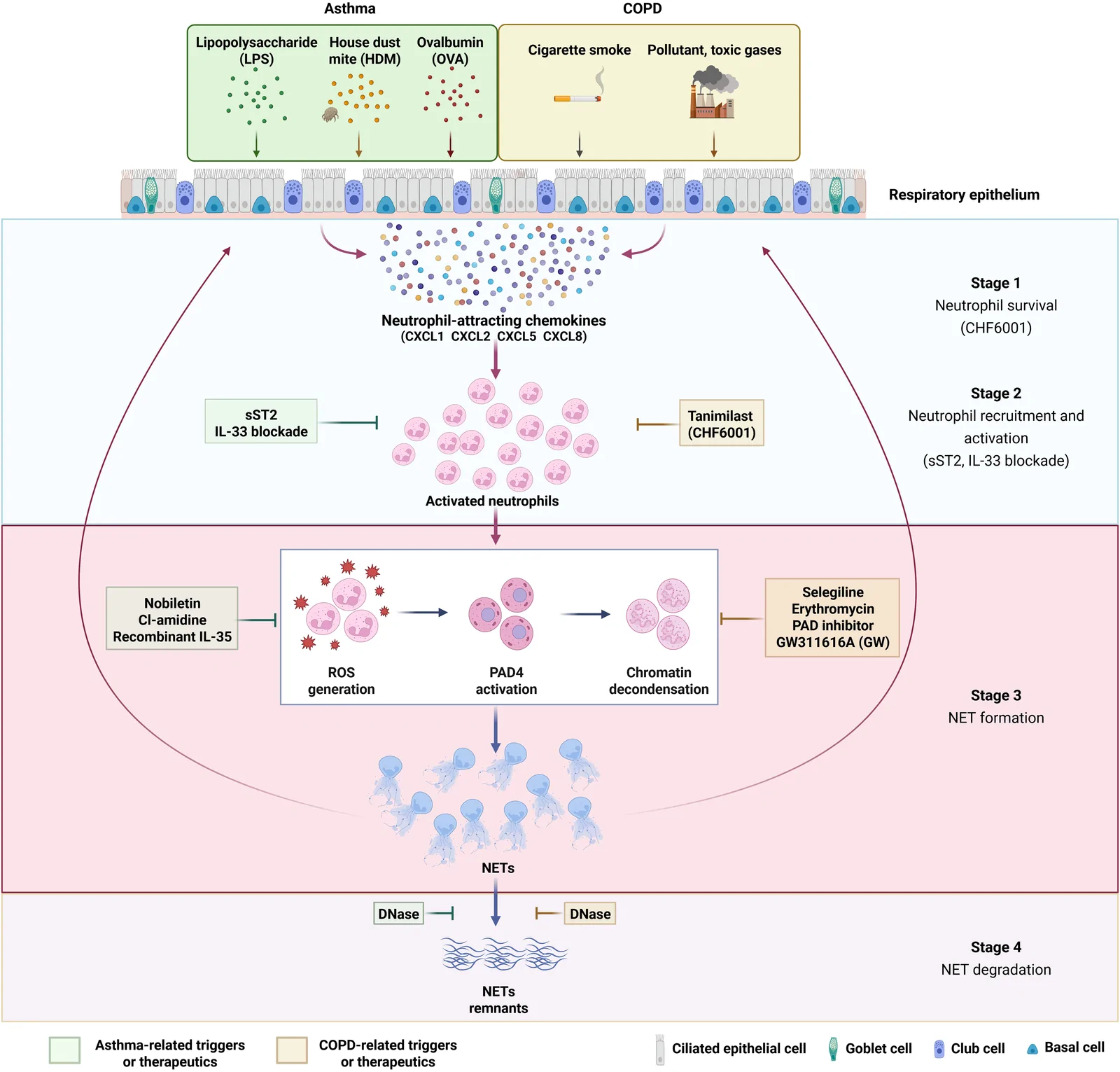
Formation and therapeutic targeting of neutrophil extracellular traps (NETs) in asthma and COPD. Airway exposure to allergens and inflammatory stimuli associated with asthma (e.g., lipopolysaccharide [LPS], house dust mite [HDM], and ovalbumin [OVA]) or to environmental exposures linked to COPD (e.g., cigarette smoke, air pollutants, and toxic gases) stimulates airway epithelial cells to release chemokines (CXCL1, CXCL2, CXCL5, and CXCL8) that promote neutrophil recruitment and activation. Activated neutrophils can form NETs via ROS production, PAD4 activation and chromatin decondensation, depending on the stimulus and NETosis pathway. Potential therapeutic approaches target different stages of this process, including (1) modulation of neutrophil survival, (2) inhibition of neutrophil recruitment and infiltration (3) suppression of NET formation, (4) enhancement of NET degradation. Green boxes show asthma-related triggers or therapeutic approaches; yellow boxes represent COPD-related triggers and therapeutic approaches. NET components can, in turn, stimulate airway epithelial cells and thereby contribute to the persistence and amplification of airway inflammation (Image created in BioRender. https://BioRender.com/5cew8co).

This review provides an overview of the main components of NETs and the molecular mechanisms underlying their formation. We further summarize current evidence on the role of NETs in asthma and COPD, discuss existing and promising NET-targeted therapeutic strategies, and examine the interplay between NETs and T helper cell responses in asthma and COPD (**Figure 1**).

## 2 Neutrophil extracellular traps

### 2.1 The components of NETs

#### 2.1.1 Nuclear DNA and mitochondrial DNA

**Figure 2:**
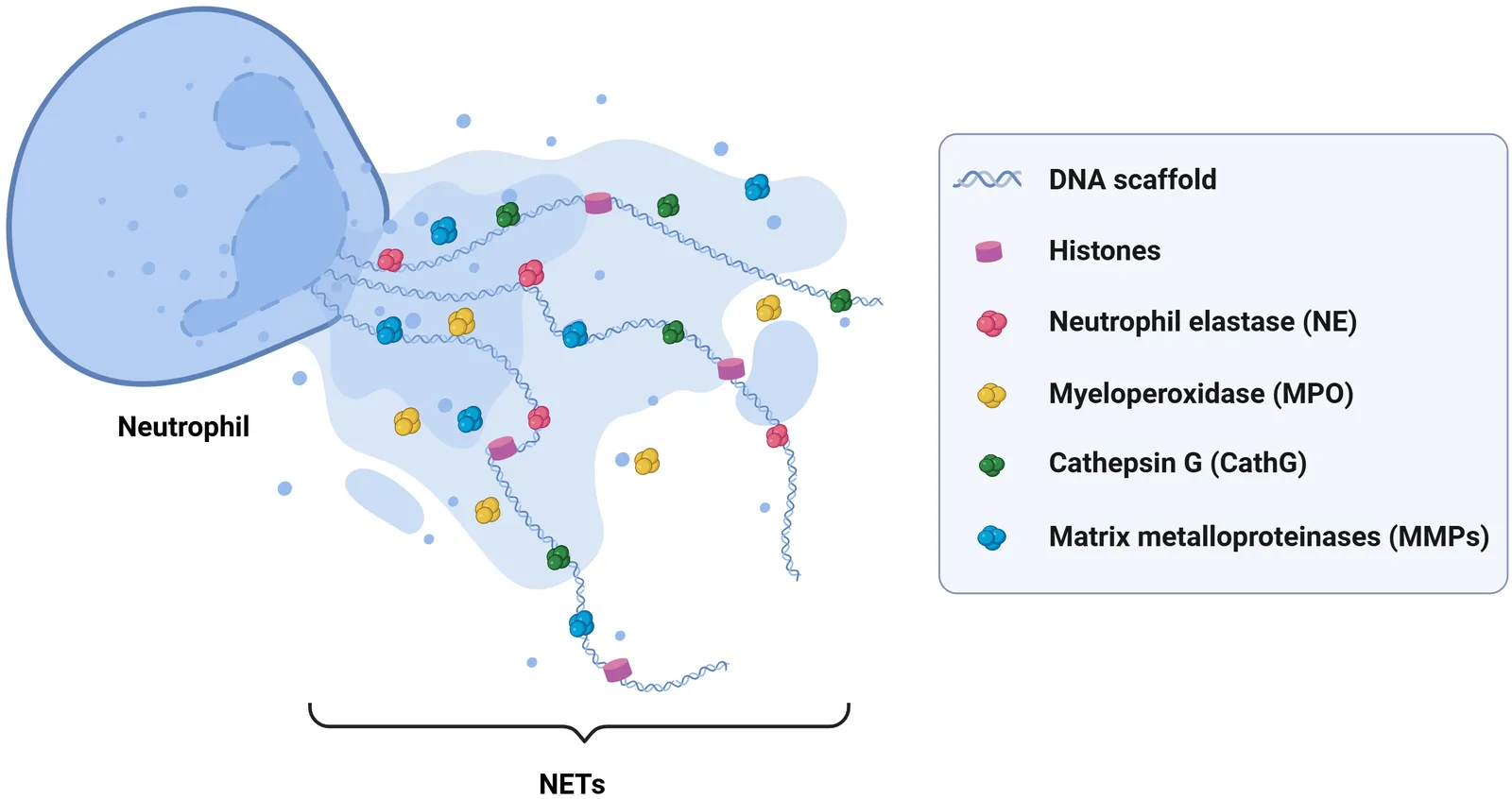
Major structural components of NETs. NETs are web-like structures primarily composed of a decondensed DNA backbone, histones, and several types of granule proteins, including neutrophil elastase (NE), myeloperoxidase (MPO), Cathepsin G (CathG) and matrix metalloproteinases (MMPs) Image created in BioRender. https://BioRender.com/9enhvqt.

The major component of NETs is filamentous DNA formed by decondensed nuclear chromatin. It serves as a net-like structural backbone and can originate from nuclear DNA, mitochondrial DNA (mtDNA), or both depending on the biological contexts. The two sources of DNA in NETs mainly show up in two distinct mechanisms of NET formation (23). In classical lytic NETosis triggered by stimuli such as bacterial pathogens, nuclear chromatin is decondensed and finally released into extracellular space with other granular proteins, causing the death of neutrophils. In non-lytic NETosis, neutrophils expel DNA without losing cell viability, a process that involves mechanistically distinct pathways and that has different requirements for reactive oxygen species (ROS). While other forms of NETosis are independent of ROS, mitochondrial DNA-mediated NET release requires the production of mitochondrial ROS (**Figure 2**).

#### 2.1.2 Histones

The other major components of NETs are five different families of histones, including H1, H2A, H2B, H3 and H4. Histones H2A, H2B, H3 and H4 account for about 70% of proteins in NETs (24). The nucleosome core is composed of two H2A–H2B heterodimers and a central H3–H4 tetramer thus building an octamer. Peptidyl arginine deiminase 4 (PAD4) mediates histone H3 citrullination through converting arginine into citrulline (25). Citrullinated histone H3 (CitH3) is used as a marker for NETosis (26, 27). Histones show various immunological characteristics including antimicrobial activity, cytotoxicity and immunomodulation (28).

#### 2.1.3 Granular proteins

Upon activation, neutrophils release several granular proteins (29) that decorate NETs. These include neutrophil serine proteases (NSPs) (30), such as NE, together with MPO, and MMPs. NE is the major protease in primary granules, which cleaves several proteins, including elastin, collagen, and fibronectin (31, 32) and exerts antimicrobial properties (33). Moreover, NE release leads to extracellular matrix degradation and further causes tissue damage and inflammation. MPO plays an important role in the oxidative burst of neutrophils and mediates bacterial killing via production of hypochlorous acid (HOCI) (34). Burn et al. proposed that MPO converts chromatin into an immune effector structure (35). NE and MPO exert synergistic effects during NET formation. ROS promote the release of NE and MPO from primary granules into the cytosol. Cytosolic NE mediates actin filament degradation before translocating to the nucleus, where it cleaves histones and initiates chromatin decondensation. Subsequent binding of MPO to chromatin further promotes this decondensation (36, 37). CathG is a serine protease belonging to the chymotrypsin-like (S1) family and is involved in immune cell regulation (38). It is predominantly expressed in neutrophils (39) and contributes to neutrophil migration through activation of metalloproteases and cleavage of extracellular matrix proteins (40). MMPs are proteolytic enzymes involved in extracellular matrix remodeling (41). Mulet et al. demonstrated that MMP-9, a component of tertiary granules, is associated with NETosis in malignant pleural effusion (MPE) (42). The above-mentioned molecules function as highly pro-inflammatory damage-associated molecular patterns (DAMPs) (43), thereby contributing to tissue injury, inflammatory cell infiltration, and amplification of inflammatory responses.

### 2.2 Mechanisms of NET formation

The mechanisms underlying NET formation have been comprehensively reviewed before (22, 44–46). Here, we briefly summarize two major forms of NETosis, lytic and non-lytic NETosis, as well as a distinct mtDNA-driven mechanism, which predominantly occurs under vital conditions (46) (summarized in **Table 1**).

**Table 1:** Mechanisms of NET formation.

Features	Lytic NETosis	Non-lytic/vital NETosis	
Cell viability	Lost	Preserved, at least initially	Generally preserved
DNA source	Nuclear	Nuclear	Mitochondrial
Stimulus	Often ROS-dependent	Pathway/stimulus-dependent	Pathway/stimulus-dependent
PAD4	Stimulus-dependent	Stimulus-dependent	Variable/context-dependent
Major extracellular components	DNA + histones + granule proteins	DNA + associated proteins	mtDNA ± associated proteins
Immediate Pathologic consequences	Tissue injury, protease/histone-mediated inflammation	NET-mediated immune modulation while retaining neutrophil functions	Potential innate immune activation by mtDNA/DAMP signaling

#### 2.2.1 Lytic NETosis

Lytic NETosis represents a distinct form of programmed neutrophil cell death, which can be induced by stimuli such as phorbol 12-myristate 13-acetate (PMA), immune complexes (ICs), or bacterial pathogens. Upon stimulation, NET formation is initiated through activation of surface receptors, including CXC chemokine receptors (CXCRs), receptors for advanced glycation end products (RAGE), and Toll-like receptors (TLRs). Subsequently, calcium is released from the endoplasmic reticulum (ER) into the cytosol, resulting in activation of protein kinase C (PKC) and membrane blebbing of both the ER and plasma membrane. Activated NADPH oxidase (NOX) generates ROS, which facilitate activation of NE and MPO and their release from granules into the cytosol and subsequently also to the nucleus. Activated PAD4 may also translocate to the nucleus, where it mediates histone H3 citrullination and promotes chromatin decondensation together with MPO and NE. These enzymatic processes induce histone degradation and nuclear membrane disassembly, enabling the mixing of nuclear contents with cytosolic proteins and granule components to form NETs. Ultimately, rupture of the plasma membrane results in extracellular release of NETs from dying neutrophils. The contribution of PAD4 varies among NETosis pathways and appears to depend on the inducing stimulus. PAD4-mediated histone citrullination is a key mechanism promoting chromatin decondensation during many forms of nuclear NET release, whereas its role in non-lytic mitochondrial NETosis (see below) remains less well defined (47).

#### 2.2.2 Non-lytic NETosis

Non-lytic NETosis encompasses several mechanisms by which neutrophils release DNA into the extracellular space while remaining viable. These pathways can be triggered by activated platelets, *Staphylococcus aureus* (*S. aureus*) (48) and complement proteins. Compared to lytic NETosis, this type of non-lytic NETosis occurs rapidly, typically within 5–25 minutes, whereas lytic NETosis generally requires several hours. Although H3 citrullination is shared with lytic NETosis, platelet- and complement-mediated NET formation frequently proceeds independently of NADPH oxidase-derived ROS.

One mechanistically distinct form of non-lytic NETosis involves the extracellular release of mtDNA. This process can be induced by stimuli such as lipopolysaccharide (LPS), C5a, IL-8, or traumatic injury (23, 46), and typically occurs within 30 minutes (49). In contrast to platelet- and complement-mediated forms of non-lytic NETosis, this mechanism requires mitochondrial ROS production and can be detected using mitochondria-specific probes such as MitoSOX dye (50). In breast cancer lung metastasis, aged neutrophils generate mtDNA-containing NETs through increased permeability of mitochondrial pore channels (51). After stimulation with *Leishmania infantum*, human neutrophils release mainly mtDNA-containing NETs (52).

In systemic sclerosis (SSc), oxidized mtDNA derived from patient plasma, together with CXCL4, promotes further mtDNA release from both neutrophils and platelets, highlighting a potential pathogenic role of mtDNA in NETosis (47, 53).

### 2.3 Consequences of lytic and non-lytic NETosis in airway disease

Lytic NETosis, through the extracellular accumulation of histones and neutrophil granule proteins, may contribute directly to epithelial injury, mucus abnormalities and amplification of local inflammation, whereas non-lytic NET release may allow neutrophils to retain effector functions while modifying the extracellular inflammatory milieu. In contrast, mtDNA-containing NETs may provide additional inflammatory signals through recognition of extracellular mitochondrial DNA as a DAMP. Thus, the biological consequences of NET formation likely depend not only on the amount of NETs generated but also on the mechanism and molecular composition of the released extracellular traps.

### 2.4 Upstream cellular and metabolic regulators of NET formation

Autophagy is a mechanism to degrade and recycle dysfunctional cellular components via the *de novo* formation of autophagosomes and their fusion with lysosomes (54). Autophagy is also induced upon nutrient starvation and has been implicated as a context-dependent upstream regulatory process that facilitates NET formation in response to specific stimuli, including aging of neutrophils (55), PMA (56), bacteria-derived peptides (57), CXCL12 (58), photobiostimulation (59) and the foodborne toxin bongkrekic acid (60). Depending on the stimulus, autophagy-mediated NET formation may involve mTOR or TLR4 signaling, together with autophagy-associated proteins, including ATG5 and ATG7 (57, 58, 61, 62). ROS-dependent autophagy may also influence the mode of neutrophil cell death, potentially shifting the response from NETosis toward apoptosis following exposure to anti-inflammatory compounds (63). Blocking autophagy can decrease NET formation in rheumatoid arthritis (64), systemic lupus erythematosus (65), acute respiratory distress syndrome (66) and non-T2 asthma (62).

Mitochondrial dysfunction (67, 68) can promote NET formation through increased mitochondrial ROS (mtROS) production, impaired mitochondrial quality control (69)and the release of mtDNA (70). Chen et al. demonstrated that cigarette smoke extract (CSE)-induced NETosis required mtROS (71).

Inflammasome activation, particularly NOD-like receptor protein 3 (NLRP3)-associated IL-1β signaling, facilitates NET formation in specific inflammatory contexts (72–75), while NET components may in turn activate the NLRP3 inflammasome signaling in macrophages (76), indicating bidirectional crosstalk between inflammasome signaling and NET formation (77).

Ferroptosis has recently emerged as a potential regulator of NET formation, given its association with oxidative stress and membrane lipid damage. Experimental evidence supports that NETs can induce ferroptosis in alveolar epithelial cells (78), intestinal endothelial cells (79) and smooth muscle cells (80), although it remains unclear whether ferroptosis directly triggers NET formation.

ER stress arises from the accumulation of misfolded and unfolded proteins in the ER (81), which triggers the so-called unfolded protein response (UPR) to orchestrate recovery from ER stress (82). Three ER sensors initiate and regulate UPR, including inositol-requiring enzyme 1 (IRE1), double-stranded RNA-activated protein kinase R (PKR)-like ER kinase (PERK), and activating transcription factor 6 (ATF6) (81). Increasing evidence indicates that ER stress and UPR signaling may contribute to neutrophil activation and NET formation (83, 84). Potential interactions between ER stress, ROS production, and PAD4-dependent NET formation are of particular interest, although the direct mechanistic relationship between these pathways remains incompletely defined. Furthermore, ER stress and UPR responses have also been recognized as important drivers of pathogenesis in asthma (85–94) and COPD (95, 96). However, direct experimental evidence demonstrating that ER stress promotes pathogenesis in asthma or COPD specifically through NET formation remains limited.

## 3 Interplay between NETs and T cells

**Figure 3:**
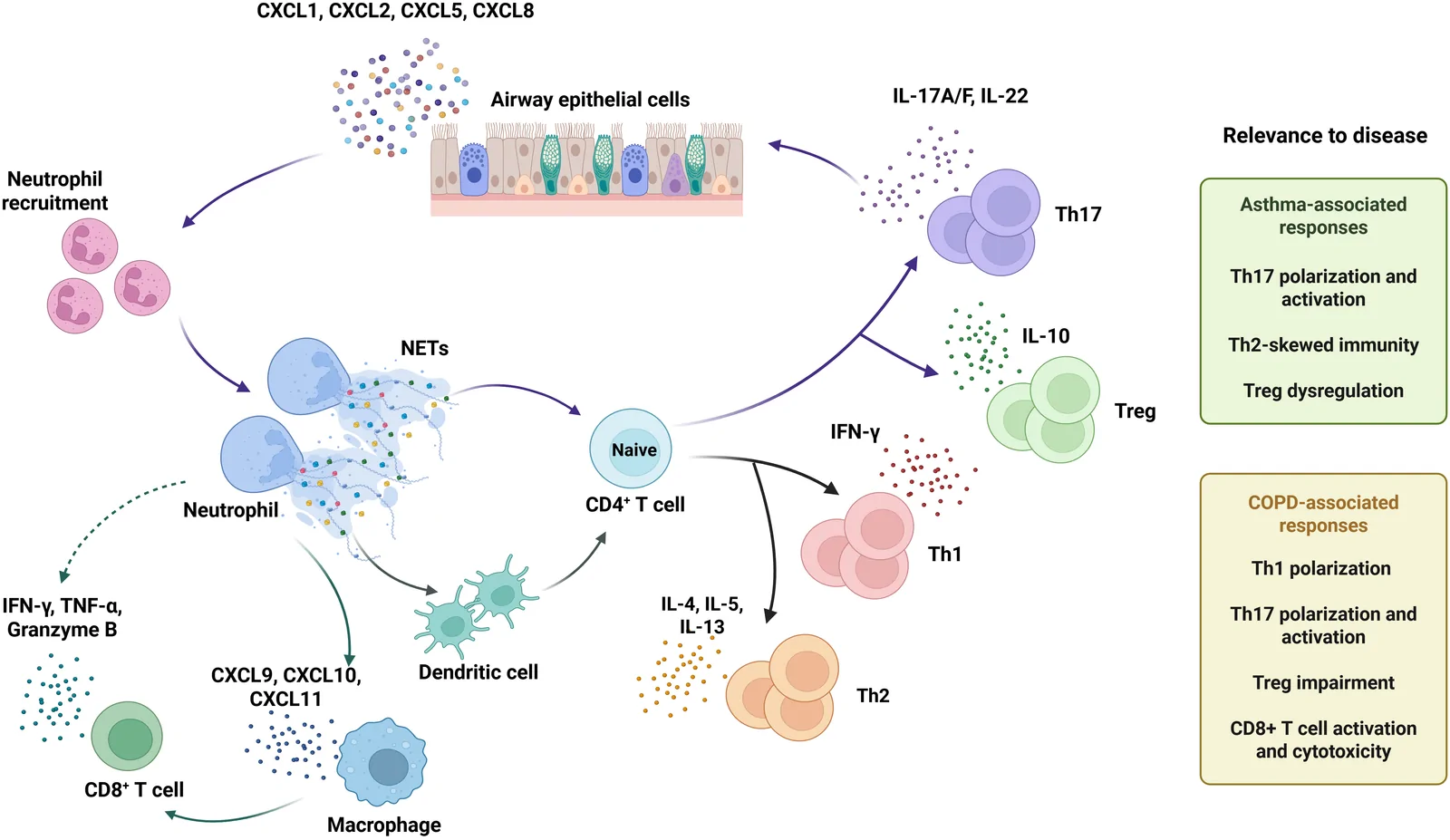
Interactions between NETs and different subpopulations of T cells. NETs influence adaptive immune responses by modulating CD4^+^ T-cell differentiation and CD8^+^ T-cell activity. NET-associated signals can promote Th1, Th2, and Th17 responses, while their effects on Treg cells are context dependent. Th17-derived IL-17A/F and IL-22 act on airway epithelial cells to induce neutrophil-recruiting chemokines, including CXCL1, CXCL2, CXCL5, and CXCL8, thereby promoting neutrophil recruitment and NET formation. NETs can also interact with dendritic cells and macrophages, which further shape T-cell responses. CD8^+^ T-cell derived signals can contribute to neutrophil activation, while macrophage-derived CXCL9, CXCL10, and CXCL11 support CD8^+^ T cell recruitment and activation. In asthma, NET-T cell interactions are particularly associated with Th17 polarization, Th2-skewed immunity, and Treg dysregulation. In COPD, these interactions have been linked to Th1 and Th17 responses, impaired Treg function, and enhanced CD8^+^ T-cell activation and cytotoxicity (Image created in BioRender. https://BioRender.com/54pekvo).

NETs link innate neutrophilic inflammation to adaptive T cell-associated responses (97) by activating antigen-presenting cells and modulating T cell differentiation and function. Emerging evidence indicates that the interactions between NETs and T cells contribute to the pathogenesis of asthma and COPD (**Figure 3**).

### 3.1 NETs and CD4^+^ T cells

A central feature of the NET–T cell axis is the ability to modulate CD4^+^ T cell polarization, particularly towards a Th17 phenotype, highlighting an important role of NETs in shaping adaptive immune responses relevant for asthma pathogenesis. NET components may act directly on T cells (98–107) or indirectly through antigen-presenting cells (108–111).

In the presence of NETs, increased frequencies of IL-17^+^ Th17 cells have been detected in peripheral blood mononuclear cells following stimulation with anti-CD3/CD28 beads (100). Through major structural components such as histones, NETs activate T cells and promote Th17 differentiation via TLR2-dependent signaling and STAT3 phosphorylation (98), thereby enhancing IL-17/Th17 responses (107). Wu et al. described a NET–CD44–IL-17A positive feedback loop in Behçet’s uveitis: NETs upregulated CD44 expression and promoted Th17 differentiation, whereas IL-17A released by Th17 cells further enhanced neutrophil recruitment and NET formation, thereby establishing reciprocal feedback loops between innate and adaptive immune responses (99). By inducing metabolic reprogramming in naïve CD4^+^ T cells, NETs have also been shown to promote polarization towards regulatory T cells (Tregs) (112, 113). Conversely, NETs can impair Treg differentiation in some inflammatory diseases (101, 104), suggesting context-dependent effects and potential therapeutic relevance of the NET–Treg axis. In addition, NETs can regulate multiple T helper cell subsets: For instance, Cathelicidin and histones promote Th17 differentiation while suppressing Treg function (106).

NETs also activate dendritic cells (DCs), macrophages (109) and other antigen-presenting cells, promoting the production of cytokines such as IL-6, IL-17, IL-1β, and IL-23 (114), that favor T-helper cells differentiation. NETs induced by free fatty acids (FFA) (108) or CSE (110) activate DCs and subsequently promote differentiation of naïve CD4^+^ T cells into Th1, Th2, and Th17 subsets. NETs have been shown to induce IFNγ-producing Th1 cells in co-cultures with mononuclear cells (115). In turn, cytokines derived from T helper cells can regulate neutrophil recruitment, activation, and NET formation.

### 3.2 NETs and CD8^+^ T cells

CD8^+^ T cells, also referred to as cytotoxic T lymphocytes (CTLs), play essential roles in antiviral and antitumor immunity (116, 117) and contribute to the pathogenesis of COPD where cigarette smoke impairs antiviral CD8^+^ T-cell responses (118). However, compared with CD4^+^ T cells, the interaction between NETs and CD8^+^ T cells has been investigated only sparsely in airway diseases. In a Sendai virus-induced asthma model, NETs promoted the recruitment and activation of both CD4^+^ and CD8^+^ T cells, effects that were attenuated by DNase I and the PAD4 inhibitor Cl-amidine, implicating NETs in the regulation of adaptive immune responses in the inflamed airway (119). Because direct evidence in chronic airway diseases remains limited, current mechanistic understanding is largely derived from studies in cancer and other inflammatory disorders. Most of these studies indicate that NETs suppress CD8^+^ T-cell-mediated cytotoxic immunity. NETs induce apoptosis of CD8^+^ T cells (120) and degrade chemokines required for their recruitment (121) and directly impair CD8^+^ T cell function through binding to their transmembrane and coiled-coil domains 6 (TMCO6) (122). In addition, NETs contribute to cancer immunotherapy resistance by reducing CD8^+^ T cell infiltration and cytotoxic activity in cholangiocarcinoma and renal cell carcinoma (123, 124). Similarly, in lung adenocarcinoma, NETs promoted tumor progression by suppressing ferroptosis and reducing CD8^+^ T cell activity through enhanced YTHDF2-mediated SLC2A3 expression (125).

Conversely, NETs may also facilitate CD8^+^ T cell infiltration under specific inflammatory conditions. In renal fibrosis, NETs enhanced production of the T cell chemoattractants CXCL9, CXCL10, and CXCL11 by macrophages, thereby promoting CD8^+^ T cell recruitment and amplifying downstream inflammatory cascades (126). On the other hand, in experimental cerebral malaria, as well as in lupus pathogenesis, CD8^+^ T cell infiltration has been shown to induce NET formation, further supporting reciprocal crosstalk between CD8^+^ T cells and NETs (127, 128). Taken together, the available evidence indicates that NETs exert context-dependent effects on CD8^+^ T-cell responses, either suppressing cytotoxic immunity or promoting inflammatory T-cell recruitment depending on the disease setting. Although direct evidence in chronic airway diseases remains limited, these findings suggest that NET-mediated modulation of antiviral CD8^+^ T-cell responses may contribute to impaired viral clearance, persistent airway inflammation, and exacerbations in diseases such as COPD. Further studies are required to determine the relevance of these mechanisms in chronic inflammatory airway diseases.

## 4 NETs in asthma

Accumulating evidence indicates that NETs contribute significantly to asthma pathogenesis, particularly in severe neutrophilic asthma, a subtype frequently associated with corticosteroid resistance.

### 4.1 Evidence from human studies and clinical samples

Dworski et al. reported the presence of eosinophil and neutrophil extracellular traps in airways of patients with atopic asthma (129). Subsequent observational studies have reported elevated levels of NET-associated markers in different compartments of patients with severe asthma. Elevated levels of NET components have been detected in peripheral blood of children with asthma, particularly during symptomatic episodes (130). Pham et al. and Varricchi et al. reported that peripheral blood neutrophils isolated from patients with severe asthma released higher levels of NET-associated markers, including dsDNA, MPO, and MMP-9, than those from patients with non-severe asthma (131) or healthy controls (132). These findings provide evidence of enhanced NET formation in asthma but are primarily observational and do not by themselves establish a causal role for NETs in disease pathogenesis.

Several studies have further investigated associations between NET markers and clinical disease characteristics. Circulating CitH3 levels were inversely correlated with FEV1/FVC ratios in asthma patients, suggesting an association between NET formation and airflow limitation (132). Using data from the French national COBRA cohort, Granger et al. demonstrated that patients with severe asthma (GINA step 4) had significantly higher circulating NET levels than patients with mild or moderate asthma and healthy controls. Notably, circulating NET levels, independent of bacterial colonization status, were more closely associated with disease severity and poor asthma control than alveolar NET levels measured in BALF (133). In addition, extracellular DNA levels in induced sputum have also been proposed as biomarkers for assessing inflammatory activity in severe asthma (134–136). Although these studies support an association between NET-related markers and disease severity, they remain correlational and cannot establish whether increased NET formation is a cause or consequence of airway inflammation.

Mechanistic studies provide further evidence that NETs can directly modulate airway inflammatory responses. Stimulation of human bronchial epithelial cells (HBEs) and A549 cells with NETs increased the secretion of CXCL1 (in A549 cells only), CXCL2, and CXCL8 through activation of the TLR4/NF-κB signaling pathway (137). Peng et al. further demonstrated that NETs upregulate RAGE and IL-8 expression without affecting TLR4 expression while blockade with FPS-ZM1 reduced p38/ERK phosphorylation and IL-8 production in HBEs (135). These findings support a direct capacity of NETs to activate airway epithelial cells and promote chemokine production. Of note, the structural integrity of NETs did not affect the chemokine release of HBEs or A549 cells or neutrophil recruitment *in vitro* (137). Thus, these experiments provide mechanistic evidence that NETs can amplify airway inflammation, rather than merely serving as biomarkers of disease activity.

In addition to circulating NET markers, bioinformatic analyses have identified potential NET-associated biomarkers in asthma. Wu et al. used peripheral blood mononuclear cell transcriptomic datasets from children with asthma together with NET-related genes previously identified in breast cancer and identified FCGR2B, FCRL5, CCR2, and FCRL1 as potential candidates associated with NET-related pathways. However, these findings remain indirect, because the analysis was based on relatively small pediatric cohorts and did not specifically involve children with neutrophilic asthma (138). Similarly, *TNFAIP3, CXCR4*, and *PADI4* have been proposed as NET-associated biomarkers in sputum and plasma samples from adults with severe asthma (139). These studies identify candidate associations but do not establish that these genes are functionally involved in NET-mediated asthma pathogenesis.

### 4.2 Evidence from murine models

Murine models have been extensively employed to investigate the mechanistic contribution of NETs to asthma pathogenesis.

Akk et al. demonstrated the presence of NETs in lung tissue, BALF, and plasma in a Sendai virus-induced murine asthma model. Airway inflammation was attenuated in mice deficient in cysteine protease dipeptidyl peptidase I (DPPI) and in mice following treatment with DNase I or Cl-amidine (119). Toussaint et al. (140) and Curren et al. (141) reported that rhinovirus (RV) infection enhanced NET release and exacerbated airway inflammation in murine asthma models. Similarly, infection with influenza A virus strain PR8 amplified asthmatic inflammation through NET-dependent mechanisms (142).

LPS also plays an important role in NET generation in house dust mite (HDM)- or ovalbumin (OVA)-induced asthma models. Exposure to low-dose LPS increased CXCR4 expression in recruited neutrophils and promoted NET release, thereby enhancing HDM uptake by CD11b^+^Ly6C^+^ DCs in HDM-induced asthma (142). In OVA/LPS-induced asthma models, inhibition of TLR4 signaling using TAK-242, a TLR4 inhibitor, suppressed phosphorylated p65 NF-κB and reduced neutrophil count in BAL fluid, which indicated involvement of NETs via the TLR4/NF-κB pathway in neutrophilic airway inflammation (137).

Adoptive transfer studies using CD39^+^CD9^+^ interstitial macrophages (IMs) isolated from lungs of mice with neutrophilic asthma have further highlighted the role of immune cells in the regulation of NET-associated inflammation (143). Transfer of these IMs suppressed NETosis, Th17 activation, and neutrophilic airway inflammation (143). Moreover, in a severe asthma model induced by OVA, HDM, and LPS, the cholesterol-lowering statin simvastatin reduced Th17 cell frequencies and NETosis through suppression of PAD4 expression, whereas intratracheal transfer of neutrophils restored NET formation and neutrophilic inflammation (144). NETs have also been linked to multiple signaling pathways in HDM- and OVA-induced asthma models. In OVA-induced asthma, NET release upregulated NQO1 expression and amplified oxidative stress, thereby aggravating mucus hypersecretion (145).

### 4.3 Evidence from equine models

Beyond murine models, several other animal species, including rats, guinea pigs, rabbits, sheep, dogs, non-human primates, and horses have contributed substantially to the understanding of asthma pathophysiology (146, 147). Among these, equine heaves, now termed severe equine asthma, is particularly relevant because it closely recapitulates several pathological and clinical features of human asthma, including lower airway inflammation, reversible airflow obstruction, and bronchial hyperresponsiveness (148–150). Similar to human neutrophilic asthma, both T helper cells and neutrophils contribute to airway inflammation in equine asthma (151). ROS production promotes pulmonary neutrophilic inflammation (148, 152–154). In 2017, Vargas et al. demonstrated that corticosteroids reduce NET formation both *in vitro* and *in vivo*. Unexpectedly, IL-17 also inhibited PMA-induced NET formation (155), suggesting a potentially complex role for this cytokine in regulating NETosis. Subsequent studies provided further evidence that NET associated markers in BAL fluid may serve as biomarkers of equine asthma severity. Janssen et al. and Cooper et al. reported that BAL fluid levels of NET-associated markers, including cfDNA (156, 157) and MPO-DNA complexes (156), correlate with disease severity in horses with severe asthma. In contrast, NET release in moderate equine asthma was not associated with neutrophil infiltration (156) suggesting that NET formation may be more closely associated with severe disease phenotypes.

## 5 NETs in chronic obstructive pulmonary disease

Persistent neutrophilic inflammation is a hallmark of COPD and is thought to result from upregulation of neutrophil chemokines and their receptors within the airway mucosa (158). The accumulation and activation of neutrophils in the lungs promote NET release, which contributes to tissue injury, disease progression and clinical deterioration in COPD (159).

### 5.1 Evidence from human studies and clinical samples

Obermayer et al. first demonstrated the presence of NETs in sputum samples from patients with acute COPD exacerbations using confocal laser scanning microscopy and electron microscopy, thereby providing solid morphological evidence of NET formation in COPD airways (160). Subsequent studies further established NETs as potential biomarkers of disease severity by analysing sputum samples from non-smokers, smokers, and patients with COPD (111, 159, 161, 162). Elevated NET levels were associated with reduced predicted FEV1, more severe clinical symptoms, increased exacerbation frequency, and altered microbial diversity in airways (159), collectively supporting a central role for NETs in COPD pathogenesis and disease progression. In addition to elevated NET levels, Wright et al. observed increased concentrations of IL-1β, CXCL8 (IL-8), PAD4, and NLRP3 in induced sputum from COPD patients (163, 164). Furthermore, serum IL-8 levels, which have been associated with corticosteroid resistance, positively correlated with NE concentrations in COPD patients, suggesting a close relationship between NETosis and corticosteroid resistance in COPD (165).

Nicotine is one of the major pathogenic components of cigarette smoke. Both nicotine and CSE have been shown to induce NETosis in human peripheral neutrophils *in vitro*. Hosseinzadeh et al. demonstrated that nicotine induces NET formation in a dose-dependent manner through mechanisms involving nicotinic acetylcholine receptors (nAChRs) and Akt signaling (166). Because ROS-dependent release of NE and MPO from neutrophil granules represents a critical step in NET formation, further studies are required to clarify how nAChR/Akt signaling intersects with NET generation in smokers. In addition, neutrophils isolated from COPD patients exhibit increased susceptibility to CSE-induced NETosis, potentially due to mitochondrial respiratory dysfunction (71, 111). These observations indicate that cigarette smoke-induced NET formation involves oxidative and mitochondrial stress pathways. CSE-induced NETs promoted proliferation of airway epithelial cells, activation of NF-κB signaling, production of type I interferons, and maturation of dendritic cells *in vitro* (71).

### 5.2 Evidence from murine models

Cigarette smoke-exposed murine models are widely used to investigate the molecular mechanisms underlying COPD pathogenesis (167). Consistent with findings from human studies, significant increases in neutrophil infiltration and NET formation have been observed in serum, BALF, and lung tissues of smoke-induced COPD mouse models (165, 168). NETs may also provide a mechanistic link between innate and adaptive immunity in COPD. Notably, CSE-induced NETs activated plasmacytoid dendritic cells (pDCs), thereby promoting differentiation of naïve CD4^+^ T cells into Th1 and Th17 subsets (110). These findings suggest that NETs not only contribute to neutrophilic inflammation but also shape adaptive immune responses that drive disease progression.

Recent investigations demonstrated elevated concentrations of 5-hydroxyindoleacetic acid (5-HIAA) in both plasma and lung tissue of COPD mice. As a ligand for G protein-coupled receptor 35 (GPR35) (169, 170), 5-HIAA enhanced LPS-induced NET formation *in vitro* (168). Pharmacological inhibition of 5-HIAA using selegiline reduced ROS generation and NET formation, thereby improving pulmonary function and attenuating inflammatory responses in COPD mice (168), further illustrating oxidative stress as an important regulator of NET formation in COPD. Likewise, PAD4 deficiency ameliorated NET formation and improved lung parenchymal pathology in elastase-induced COPD model of *Padi4* gene knockout mice (171). Furthermore, Wang et al. reported that prolonged cigarette smoke exposure increased corticosteroid resistance in a murine COPD model through triggering ferroptosis resistance in neutrophils, which finally promoted NET formation (165). This finding may indicate the potential role of the ferroptosis-NETosis mechanism in the NET-associated pathogenesis of COPD.

## 6 Targeting NETs to treat asthma and COPD

NET-targeted therapeutic strategies in asthma and COPD can be broadly categorized into four groups based on their mechanisms of action (**Table 2**): (1) prolongation of neutrophil survival, (2) reduction of neutrophil recruitment and infiltration, (3) suppression of NET formation, and (4) enhancement of NET degradation. Collectively, these approaches are intended to mitigate NET-mediated airway inflammation and tissue injury while improving disease control.

**Table 2:** Asthma and COPD therapy by targeting NETs.

Therapeutic strategies	Treatment	Molecular target	Mechanism	Disease	Developmental stage	Limitations
prolongation of neutrophil survival	Tanimilast (CHF6001) (172, 173)	PDE4	Inhibiting PDE4-dependent signaling	COPD	clinical	Not NET-specific
inhibition of neutrophil recruitment and infiltration	IL-33 blockade (141)	IL-33/ST2 axis	Reducing IL-33-driven signaling	Asthma	preclinical and clinical depending on agents	Not NET-specific
	sST2 (62)	IL-33/ST2 axis	Limiting IL-33/ST2 signaling		preclinical	Limited clinical evidence
suppression of NET formation	Cl-amidine (119)	PAD enzymes, especially PAD4	Inhibiting histone citrullination and chromatin decondensation	Asthma	preclinical	Not specific selectivity
	Nobiletin (174)	Oxidative pathways	inhibiting oxidative signaling		preclinical	Not NET-specific
	Recombinant IL-35 (175)	IL-35-associated pathways	Suppressing neutrophil activation		preclinical	Limited clinical evidence
	Erythromycin (EM) (111)	Macrolide-associated immunomodulatory pathways	Reducing neutrophilic inflammation	COPD	preclinical (NET-targeted application)	Not NET-specific
	Selegiline (168)	Monoamine oxidase B	inhibiting oxidative stress		preclinical	Limited evidence
	GW311616A (GW) (177, 178)	NE	Suppressing NE-associated processes		preclinical	NET formation may occur through NE-independent pathways (184)
	PAD inhibitor (178)	PAD family	Inhibiting histone citrullination and chromatin decondensation		preclinical	Efficacy varies with NET stimulus
enhancement of NET degradation	DNase (119, 178, 179)	Extracellular DNA	Degrading the DNA scaffold of NETs	Asthma & COPD	preclinical	Can not inhibit NET formation and may promote release of NET proteins

Tanimilast (CHF6001), a novel inhaled phosphodiesterase-4 (PDE4) inhibitor currently under investigation for COPD treatment (172), has been shown to prolong neutrophil survival while reducing NET production (173).

Curren et al. demonstrated that IL-33 blockade ameliorated RV-induced asthma exacerbation by disrupting neutrophil recruitment and NET formation (141). In an HDM-induced asthma model combined with cigarette smoke exposure, administration of soluble ST2 (sST2), which neutralizes IL-33 in an mTOR-dependent manner, reduced pulmonary neutrophil infiltration, CD40^+^ and CD86^+^ dendritic cell populations, and Th2/Th17 responses (62).

In a murine model with neutrophilic asthma, Nobiletin, a naturally occurring polymethoxylated flavone, inhibited NET formation through suppression of PI3K/AKT and STAT3 signaling pathways (174). Recombinant IL-35 treatment similarly alleviated STAT3-mediated ferroptosis-associated NET formation and restored the DC–Th17/Treg balance (175). Similarly to the findings in asthma models, a growing body of evidence supports therapeutic targeting of NET components in COPD. The antibiotic erythromycin was found to suppress NET formation, to inhibit myeloid dendritic cell (mDC) activation, and to attenuate Th1/Th17 responses in cigarette smoke-exposed mice (111). Erythromycin further attenuated inflammation through multiple pathways, including PI3K/AKT, mTOR, NF-κB, PTEN, and oxidative stress-related signaling (176). Peritoneal administration of GW311616A, a selective NE inhibitor, reduced neutrophilic inflammation and improved lung function in a murine COPD model (177). Furthermore, in a virus-induced COPD exacerbation model, treatment with GW311616A or PAD inhibitors attenuated immunopathology, further highlighting the importance of NET-associated components in disease severity (178). Finally, DNase I treatment enhanced degradation of NETs, alleviating inflammation in murine models of asthma (119) and COPD (178, 179).

Pharmacological modulation of ER stress may offer an additional strategy for limiting NET-associated inflammation. Chemical chaperones such as 4-phenylbutyric acid (4-PBA) can attenuate pulmonary inflammatory responses and neutrophil recruitment (180). Inhibitors of individual UPR branches, including PERK (181) and IRE1α/XBP1 signaling (182, 183), have shown anti-inflammatory effects in an *in-vitro* model of lung epithelial cell line (A549) transfected with SARS-CoV-2 ORF3a mRNA (181), human bronchial epithelial cells in cystic fibrosis (182) and a murine model of acute lung injury (183). These studies pose the possibility that ER stress-related therapies may help alleviate neutrophilic inflammation via suppressing neutrophil activation and NET formation. However, direct evidence demonstrating that ER stress regulating NET formation in asthma and COPD remains limited.

### 6.1 NETosis as a driver of corticosteroid resistance in neutrophilic airway disease

Neutrophilic airway inflammation is strongly associated with reduced responsiveness to corticosteroid therapy, particularly in patients with severe asthma and COPD (185, 186), thereby limiting disease control and symptom relief. Reduced expression of histone deacetylase 2 (HDAC2) (185, 187) and increased phosphoinositide 3-kinase δ (PI3Kδ) (188, 189), are widely recognized as key molecular determinants of corticosteroid resistance, particularly in COPD.

### 6.2 NET-mediated neutrophilic inflammation

NET-associated oxidative stress may contribute to this process by activating the PI3Kδ/Akt pathway, leading to phosphorylation and functional inactivation of HDAC2. In parallel, excessive production of reactive oxygen and nitrogen species promotes post-translational HDAC2 modifications, that facilitate its inactivation (190) and subsequent proteasomal degradation (191) ultimately reducing HDAC2 abundance. As HDAC2 is essential for corticosteroid receptor-mediated suppression of pro-inflammatory gene transcription, its depletion diminishes corticosteroid efficacy. Furthermore, oxidative stress and inflammatory mediators activate p38 MAPK, impairing corticosteroid receptor nuclear translocation and transcriptional activity, thereby reinforcing corticosteroid insensitivity. In addition to directly impairing corticosteroid signaling, NETosis perpetuates inflammatory pathways that are relatively insensitive to corticosteroid-mediated suppression.

NET components activate pattern-recognition receptors, including TLR2, TLR4, TLR9 and RAGE. This results in the persistent activation of NF-κB and the continued production of pro-inflammatory mediators, such as IL-1β, IL-6, TNF-α and CXCL8 (also known as IL-8). IL-17 further amplifies neutrophilic inflammation by inducing the production of CXCL8 in airway epithelial cells (192). This promotes continued neutrophil recruitment and creates conditions that favor persistent NETosis. NET-derived proteases stimulate epithelial cells and resident macrophages to release CXCL8, which recruits additional neutrophils via CXCR1/CXCR2 signaling, while also delaying neutrophil apoptosis. The prolonged survival of activated neutrophils increases the likelihood of further NET formation, thus establishing a self-perpetuating inflammatory circuit. It is noteworthy that corticosteroids themselves may paradoxically prolong neutrophil lifespan by inhibiting apoptosis, thereby potentially amplifying this pathogenic feedback loop in neutrophilic airway disease.

Collectively, these findings suggest that NETosis is not merely a consequence of persistent airway inflammation but may actively reinforce corticosteroid resistance by promoting oxidative stress, impaired corticosteroid receptor signaling, and sustaining neutrophil recruitment and survival. Although direct causal evidence remains limited, accumulating experimental and clinical data support the concept that targeting NET formation or its downstream signaling pathways may improve corticosteroid responsiveness in selected patients with neutrophilic asthma and COPD.

### 6.3 Therapeutic implications

The recognition that NETosis contributes to corticosteroid resistance has important therapeutic implications for neutrophilic asthma and COPD. In addition to directly targeting NET formation or promoting NET degradation, strategies aimed at interrupting the molecular pathways that link NETosis to corticosteroid insensitivity may enhance treatment efficacy. This could involve inhibiting CXCL8/CXCR1/CXCR2 signaling to limit neutrophil recruitment, suppression of oxidative stress to preserve HDAC2 activity, and modulation of PI3Kδ signaling to restore corticosteroid receptor function. Furthermore, therapies with anti-neutrophilic properties, such as macrolides and PDE-4 inhibitors, have demonstrated clinical benefit in selected patients, primarily through reducing neutrophilic inflammation and exacerbation frequency (193–196). While most of these approaches remain investigational or are supported by limited clinical evidence, they emphasize the potential of combining conventional corticosteroids with therapies that target NET-associated pathways to improve outcomes in cases of corticosteroid-resistant neutrophilic airway disease.

## 7 Current research gaps

### 7.1 Limited understanding of NET heterogeneity

NETs display substantial heterogeneity depending on the initial stimulus and the type of DNA involved, including nuclear DNA and mitochondrial DNA. As discussed in Section 2.2, nuclear DNA is primarily involved in lytic NETosis (44), whereas mitochondrial DNA contributes to a specialized form of non-lytic NETosis (46). These can be distinguished using mitochondrial markers in immunofluorescence staining and microscopy (49, 50). However, many studies do not verify the DNA origin of NETs, resulting in the loss of potentially important mechanistic information. NET heterogeneity is further increased by the diversity of neutrophil subsets and activation states. Depending on the pathological context, NETs may be formed by suicidal neutrophils during antimicrobial defense (197), by non-lytic NETosis in response to *S. aureus* (48), by immature or low-density neutrophils in COVID-19 and sepsis (198), by tumor-associated neutrophils in cancer (199, 200), by aged neutrophils during metastatic progression (51, 201), or by neutrophil-platelet aggregates in thromboinflammatory diseases (202, 203). Importantly, although NET formation is frequently mediated through ROS-dependent and PAD4-associated pathways (204, 205), ROS- (206, 207) or PAD4-independent (208) mechanisms have also been described in specific pathological contexts. A more refined classification framework integrating NET composition, subpopulation origin, and signaling pathways will be essential for defining biologically distinct NET subtypes and understanding their disease-specific functions.

Emerging technologies may provide a more detailed view of neutrophil heterogeneity and NET biology. Single-cell transcriptomic approaches are increasingly used to identify distinct neutrophil states in inflamed airways and lung tissues. CXCR4^hi^ neutrophils were identified as early inflammatory triggers in mouse models of allergic asthma in the lungs (142). In a cigarette smoke-exposed murine model of COPD, Kapellos et al. showed that murine BALF and blood neutrophil phenotypes shared transcriptional similarities with human ones isolated from BALF (209). Furthermore, spatial transcriptomic approaches can map the distribution of neutrophil populations in disease-associated cellular niches (210, 211). By integrating single-cell and spatial transcriptomic data, Chen et al. constructed a cross-etiology atlas of acute lung injury that revealed etiology-dependent neutrophil fate decisions and inflammatory crosstalk between neutrophils and macrophages (212). Similarly, in a golden hamster model of SARS-CoV-2 infection, Yang et al. used single-cell spatiotemporal mapping to trace the migration of splenic neutrophils to the lung during the peak antiviral response (213). These approaches may help link specific neutrophilic states and tissue microenvironment to NET formation.

### 7.2 Lack of standardized methods for NET detection

NETs are commonly identified using markers such as cfDNA, MPO–DNA complexes, CitH3, NE, or colocalization of multiple markers by immunofluorescence staining (129, 214) or flow cytometry (215, 216). Although widely used, these methods remain susceptible to false-positive interpretation. On the one hand, cfDNA and granule-associated proteins may originate from alternative cell death pathways independent of NETosis or from dying cells other than neutrophils (217, 218). On the other hand, antibody specificity remains a significant technical challenge. Thålin et al. developed an assay to quantify CitH3 in human plasma and found that several commercially available anti-CitH3 antibodies also cross-reacted with non-CitH3 (27). The development of standardized and validated methods for NET detection is therefore essential to improve reproducibility enabling cross-study comparisons and accelerating translational research.

### 7.3 Limitations in translating murine models to human disease

Species-specific differences between murine and human neutrophils represent a major obstacle for translating findings from animal models into clinical applications. Human and murine neutrophils differ in nuclear morphology, receptor expression, granule protein composition, and cytokine production (219). More importantly, recent reviews have highlighted inherent limitations of preclinical studies targeting NETs, including the inability of mouse models to fully recapitulate the complexity and temporal dynamics of human disease as well as species-specific differences in NET-forming capacity (220, 221). Consequently, NET-associated pathways identified in murine models may not fully reflect human pathophysiology. Future studies integrating patient-derived samples, organoid systems, lung-on-chip platforms and *ex-vivo* human neutrophil models will be essential for strengthening translational relevance and bridging the gap between mechanistic discoveries and clinical application.

### 7.4 Challenges and future perspectives of NET-targeted therapies

Despite considerable progress in elucidating the biological functions of NETs, their translation into clinically effective therapeutic strategies remains challenging. Because NETs constitute an evolutionarily conserved component of innate immunity that contributes to pathogen trapping and microbial killing (19, 222) indiscriminate or sustained systemic inhibition of NET formation may impair physiological immune functions and increase susceptibility to bacterial, fungal, and potentially viral infections (223).

The dual role of NETs as both protective and pathogenic structures highlights the need to balance pathogen clearance with the prevention of excessive tissue injury (224). While excessive or persistent NET formation promotes endothelial dysfunction (225), thrombosis (226, 227), chronic inflammation (228), and autoantigen exposure, complete suppression of NET generation could compromise microbial clearance and delay the resolution of infection (36, 229). Therefore, future therapeutic strategies should aim to selectively attenuate pathological NET formation while preserving their physiological antimicrobial functions.

Several studies have demonstrated disease-specific differences in NET formation and clearance in systemic lupus erythematosus (230), ANCA-associated vasculitis (231), rheumatoid arthritis (232), and thrombosis (226) and may further depend on disease stage, inflammatory milieu, and genetic background (36). These observations suggest that NET burden varies substantially among patients and that only selected patient populations are likely to benefit from NET-directed therapy, emphasizing the need for improved patient stratification and precision medicine approaches.

Reliable biomarkers are therefore essential for patient stratification and treatment monitoring. Several original studies have identified circulating MPO-DNA complexes, neutrophil elastase-DNA complexes, citH3, and impaired DNase-mediated NET degradation as indicators of disease activity (231, 233, 234). Nevertheless, none of these biomarkers alone possesses sufficient specificity or standardization for routine clinical implementation, suggesting that future biomarker panels integrating multiple NET-associated parameters may provide greater diagnostic and prognostic accuracy.

Finally, despite encouraging preclinical findings, relatively few NET-targeting compounds have progressed into clinical evaluation (235, 236) with variable therapeutic efficacy. Current approaches include degradation of extracellular NETs by recombinant DNase I, inhibition of PAD4, neutrophil elastase inhibition, blockade of gasdermin D-mediated pore formation, and modulation of upstream inflammatory pathways such as CXCR2 or complement signaling. However, many of these strategies remain limited by incomplete target specificity, off-target effects, uncertain long-term safety, suboptimal pharmacokinetic properties, and the absence of validated clinical endpoints that directly reflect NET inhibition.

Future research should therefore focus not only on developing more selective inhibitors of pathological NET formation but also on defining disease-specific therapeutic windows, validating biomarker-guided treatment strategies, and identifying patients most likely to benefit from NET modulation. Such precision medicine approaches may enable the therapeutic exploitation of NET biology while minimizing disruption of essential host defense mechanisms.
