# Correction: Neutrophil extracellular traps in asthma and chronic obstructive pulmonary disease: pathogenic roles, and therapeutic opportunities

**DOI:** 10.3389/fimmu.2026.1993356

**Published:** 2026-10-05

**Authors:** Xijie Zhang, Tobias Dallenga, Susanne Krauss-Etschmann

**Affiliations:** 1 Division of Early Life Origins of Chronic Lung Diseases, Research Center Borstel (RCB), Airway Research Center North (ARCN), German Center for Lung Research (DZL), Borstel, Germany; 2 Division of Cellular Microbiology, Research Center Borstel-Leibniz Lung Center, Borstel, Germany; 3 German Center for Infection Research (DZIF), Partner Site Hamburg-Lübeck-Borstel-Riems, Borstel, Germany; 4 DZL-Laboratory for Experimental Microbiome Research, Research Center Borstel, Airway Research Center North (ARCN), German Center for Lung Research (DZL), Borstel, Germany; 5 Institute of Experimental Medicine, Christian-Albrechts University Kiel, Kiel, Germany

**Keywords:** asthma, chronic obstructive pulmonary disease (COPD), neutrophil extracellular traps (NETs), T cells, therapy

The **Funding** from the German Academic Exchange Service (DAAD) to Xijie Zhang was erroneously omitted. The work was made possible with the support of a scholarship from the German Academic Exchange Service (DAAD).

The original version of this article has been updated.

The BioRender link in the legend of **Figure 3** was incorrectly published as “https://BioRender.com/54pekv”. The correct link is “https://BioRender.com/54pekvo”.

A correction has been made to the legend of **Figure 3**:

“Image created in BioRender. https://BioRender.com/54pekvo.”

The original version of this article has been updated.

